# Phase II Study Combining Pembrolizumab with Aromatase Inhibitor in Patients with Metastatic Hormone Receptor Positive Breast Cancer

**DOI:** 10.3390/cancers14174279

**Published:** 2022-09-01

**Authors:** Xuan Ge, Susan E. Yost, Jin Sun Lee, Paul H. Frankel, Christopher Ruel, Yujie Cui, Mireya Murga, Aileen Tang, Norma Martinez, Samuel Chung, Christina Yeon, Daphne Stewart, Daneng Li, Swapnil Rajurkar, George Somlo, Joanne Mortimer, James Waisman, Yuan Yuan

**Affiliations:** 1 Department of Medical Oncology & Therapeutics Research, City of Hope Comprehensive Cancer Center, Duarte, CA 91010, USA; 2 Department of Statistics, City of Hope Comprehensive Cancer Center, Duarte, CA 91010, USA; 3 Cedars-Sinai Cancer, Los Angeles, CA 90048, USA

**Keywords:** pembrolizumab, aromatase inhibitor, metastatic, hormone receptor positive, breast cancer

## Abstract

**Simple Summary:**

Aromatase inhibitors (AIs) remain key elements of endocrine therapy for patients with hormone receptor positive HER2 negative metastatic breast cancer (HR^+^ HER2^−^ MBC). Recent advances include the use of CDK 4/6 inhibitors or PIK3CA inhibitor in the clinic; however, few immunotherapy trials have been conducted in HR^+^ HER2^−^ metastatic disease. The current phase II trial was designed to test the safety and efficacy of AIs in combination with immune checkpoint inhibitor pembrolizumab in patients with HR^+^ HER2^−^ MBC (NCT 02648477). This combination was well tolerated, but minimal clinical activity was observed likely due to lack of PD-L1 pre-selection and heavily pretreated patient population.

**Abstract:**

This study investigated the safety and antitumor activity of aromatase inhibitors (AI) with immune checkpoint inhibitor (ICI) pembrolizumab in patients with hormone receptor positive (HR^+^) human epidermal growth factor receptor 2-negative (HER2^−^) metastatic breast cancer (MBC) in a phase II study with a safety lead-in (NCT 02648477). Patients received pembrolizumab plus AI up to 2 years or until confirmed progression or unacceptable toxicity. Key eligibility criteria were HR^+^ HER2^−^ MBC; RECIST v1.1 measurable disease; adequate organ function; and ECOG 0-1. Primary endpoints were safety and overall response rate. A 3-at-risk design was used for the safety lead-in with a targeted accrual of 20 patients. Grade 2 adverse events (AEs) included 35% fatigue, 20% rash, and 10% hot flashes. Grade 3 immune-related AEs (irAEs) related to pembrolizumab included 5% elevated AST/ALT, 5% rash, and 5% lymphopenia. Two (10%) patients had partial responses, three (15%) had stable disease, and 15 (75%) had progression of disease. Median progression-free survival was 1.8 months (95% CI 1.6, 2.6), median overall survival was 17.2 months (95% CI 9.4, NA), and median follow-up time was 40.1 months (range 31.3–46.8 months). The combination was well tolerated, but clinical activity was comparable to AI alone.

## 1. Introduction

Hormone receptor positive (HR^+^) human epidermal growth factor receptor 2-negative (HER2^−^) breast cancer (BC) accounts for approximately 60% of all breast cancers [1]. Despite multiple currently available treatment modalities such as selective ER modulators (SERMs), aromatase inhibitors (AIs), and selective ER degraders (SERDs), both alone or in combination with cyclin-dependent kinase 4/6 inhibitors (CDK4/6i), mammalian target of rapamycin (mTOR) inhibitors, or PIK3CA inhibitor, HR^+^ MBC patients eventually develop endocrine resistance and are considered incurable [1,2,3,4,5,6,7]. Thus, there is an unmet need to discover novel treatment strategies that may overcome innate or acquired endocrine resistance.

The immune checkpoint inhibitor (ICI) pembrolizumab in combination with chemotherapy has shown significant progression-free survival (PFS) benefit for first-line therapy of metastatic triple-negative breast cancer (mTNBC) observed in the KEYNOTE 355 trial. In the KEYNOTE 522 trial, addition of pembrolizumab to chemotherapy significantly increased pathological complete response (pCR) and prolonged event-free survival (EFS) in patients with high-risk early-stage TNBC [8,9,10]. However, the role of ICIs is not well established in HR^+^ metastatic breast cancer (MBC). 

HR^+^ HER2^−^ BC is well known for its lack of tumor infiltrating lymphocytes (TILs) and PD-L1 expression and is considered “immune-cold.” Approximately 10–15% of ER^+^ HER2^−^ BC expresses PD-L1 detected by 22C3 antibody testing [11]. Preclinical evidence has shown that high ER levels decrease PD-1/PD-L1 expression and CD8^+^T cell infiltration by suppressing Th17 cell infiltration and IL-17 signal transduction in breast cancer [12]. Modulation of host immune and tumor microenvironment (TME) may be the key to effective immune responses in HR^+^ HER2 MBC. Due to potentially synergistic effects and non-overlapping toxicities, there is good rationale to assess the combination of pembrolizumab and AI in patients with HR^+^ HER2^−^ MBC. The current trial was designed to evaluate the safety and efficacy of AI and pembrolizumab. 

## 2. Materials and Methods

Clinical trial: This phase II trial for patients with metastatic hormone receptor positive (HR^+^) BC was conducted at the City of Hope National Cancer Center between March 2016 and October 2019 with the approval of the institutional review board (IRB), the World Medical Association’s Ethical Principles, the International Conference on Harmonization of Technical Requirements, Good Clinical Practice guidelines, and U.S. federal regulations. Informed voluntary consent was signed by all patients prior to study entry (NCT02648477). HR^+^ patients received pembrolizumab 200 mg IV every 3 weeks, and anastrozole (1 mg), letrozole (2.5 mg), or exemestane (25 mg) daily until progression. 

Eligibility criteria: Patients were eligible if they were 18 years or older and had metastatic HR^+^ BC defined by ASCO/CAP guidelines, measurable disease based on RECIST 1.1, a performance status of 0 or 1 on the Eastern Cooperative Oncology Group (ECOG) performance scale, life expectancy of 3 months or more, and adequate organ function. Prior exposure to aromatase inhibitor, fulvestrant, CDK4/6i, everolimus, or chemotherapy were not excluded, unless patient was deemed to be resistant to all three (anastrozole, letrozole, exemestane) approved AIs (defined as progression within 12 months or while on an AI).

Study objectives: Objectives included evaluation of overall response rate (ORR) of the combination of pembrolizumab and oral aromatase inhibitor in patients with stage IV HR^+^ HER2^−^ BC, and assessment of clinical benefit rate (CBR) (no progression for >24 weeks), progression-free survival (PFS), and overall survival (OS) based on RECIST 1.1 and irRECIST. In addition, safety and tolerability were assessed based on toxicities by CTCAE 4.0. 

Best response: CT scans were performed at baseline and every 9 weeks after the first dose, and patients with complete response (CR) or partial response (PR) were confirmed by a second examination 4 weeks or more later. Patients were deemed to have stable disease (SD) if one or more post-treatment assessments of SD were observed 8 weeks after treatment started. 

Statistical analysis: During the safety lead-in, at most three patients were allowed to be at risk for first cycle toxicities at any one time [13]. When the first six patients completed cycle 1 with at most one dose limiting toxicity (DLT), the safety-lead in for the doublet was considered successful, and accrual proceeded based on the Phase II considerations. If two DLTs were observed on dose level 1 in the first six patients, the study would hold accrual for the selection of a dose level −1 or termination of study. For patients who recurred on or after, or progressed on prior endocrine therapy, objective response in this setting was rare for AI (~10%) [14,15]. With 20 patients, if three or more patients experienced a response, this was considered worthy of further evaluation. This rule had a power of over 80% to detect a true response rate of 20% (encouraging, alternative hypothesis) and a type I error of less than 8% for falsely declaring a 5% response rate (discouraging, null hypothesis) as promising. This rule established the minimum response rate for declaring the combination promising; however, it did not preclude that the combination could be promising based on secondary endpoints such as progression-free survival. Analysis by prior treatment may also influence the overall recommendation. Kaplan–Meier analysis was used for clinical outcomes, and median follow-up was calculated using reverse Kaplan–Meier. The 95% CI for ORR and CBR were calculated using the Clopper–Pearson method.

TIL analysis: Stromal TILs were measured by hematoxylin and eosin (H&E) staining of formalin-fixed paraffin-embedded (FFPE) tumor biopsies according to the International TIL Work Group guideline [16]. 

PD-L1 analysis: QualTek Molecular Laboratory (Goleta, CA, USA) performed PD-L1 on FFPE tumor sections from baseline testing using 22C3 antibody (Merck & Co., Kenilworth, NJ, USA) for immunohistochemistry (IHC). PD-L1 positivity was defined as membrane staining in at least 1% of cells [17,18,19].

## 3. Results

### 3.1. Patient Characteristics

Twenty eligible patients were accrued from March 2016 to October 2019 and received 200 mg pembrolizumab IV (every 3 weeks) plus AI. The median age was 62 years (34–79 years); 15 (75%) were Caucasian, 3 (15%) Asian, 1 (5%) Pacific Islander, and 1 (5%) unknown (Table 1). Major sites of metastasis were bone 90%, liver 60%, and lung 20%. Median lines of therapy were 2 (0, 7). Fourteen (70%) patients had prior CDK4/6 inhibitor (CDK4/6i), 15 (75%) patients received prior fulvestrant, 14 (70%) received prior AI (18 patients had fulvestrant and/or AI), and seven (35%) received prior everolimus.

### 3.2. Efficacy

**Figure 1:**
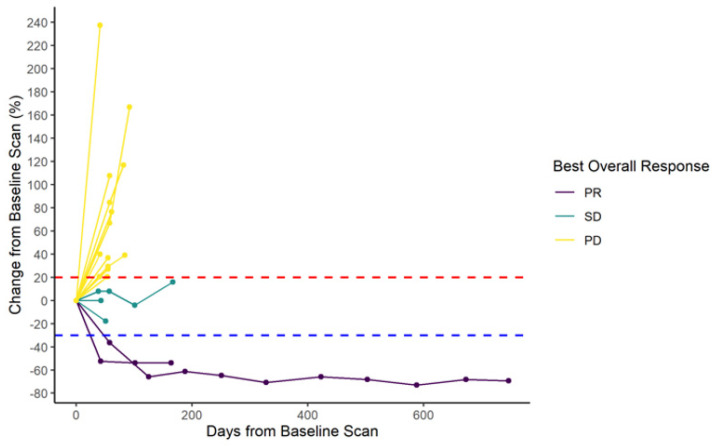
Response to AI plus pembrolizumab (N = 20). Spider plot showing relative change in tumor size from baseline. Each line represents one patient in study. Two patients had partial response (PR), three had stable disease (SD), and 15 had progression of disease (PD). One patient had sustained PR until end of treatment (35 cycles) of pembrolizumab plus exemestane and maintained response on single agent exemestane until data cut-off of July 2021. Red line indicates 20% of increased tumor size per RECIST 1.1. Blue line indicates 30% of reduced tumor size per RECIST 1.1.

**Figure 2:**
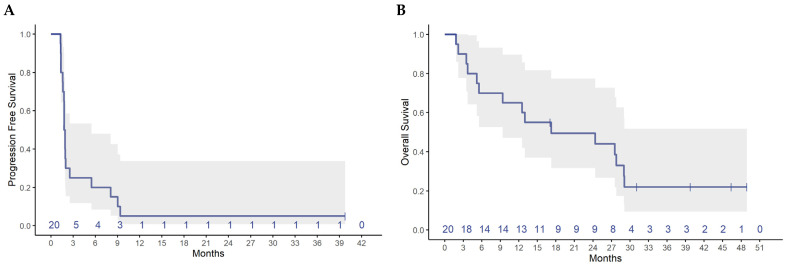
Progression-free survival and overall survival (N = 20). (**A**) Median PFS was 1.8 months (95% CI 1.6, 2.6), and (**B**) median OS was 17.2 months (95% CI 9.4, NA). Numbers above x-axis refer to number of patients.

Treatment with AI plus pembrolizumab resulted in a clinical benefit rate (CBR) at 6 months of 20% (95% CI 5.7, 43.7) and an overall response rate (ORR) of 10% (95% CI 0.3, 44.5) (Table 2). Two (10%) patients had PR, three (15%) patients had SD, and 15 (75%) patients had PD as best response. The spider plot shows relative changes in tumor size from baseline over time (Figure 1). One patient had sustained PR until end of treatment (35 cycles) of pembrolizumab plus exemestane and maintained response on single agent exemestane until data cut-off of July 2021. Median follow-up time was 40.1 months (range 31.3–46.8 months). Median PFS was 1.8 months (95% CI 1.6, 2.6), and median OS was 17.2 months (95% CI 9.4, NA) (Figure 2).

The first partial responder was a 61-year-old woman with de novo stage IV ER^+^ PR^+^ HER2^−^ metastasis to bone and lung, PD-L1 negative by 22C3. This patient received exemestane and pembrolizumab in this study as front-line treatment for metastatic disease. Initial diagnosis was February 2017. The patient started C1D1 on 7 March 2017 and achieved PR by RECIST 1.1 after 12 weeks (four cycles) of treatment. PR was sustained until end of treatment (35 cycles) of pembrolizumab plus exemestane. The patient maintained response until data cut-off of July 2021 while on single agent exemestane.

The second partial responder was a 67-year-old woman with stage IV ER^+^ PR^+^ HER2^−^ primary breast cancer with metastasis to lymph node, bone, lung, and liver with two prior lines of treatment (fulvestrant and letrozole/palbociclib) for metastatic disease. The patient initially was diagnosed in December 2006 with left-sided stage IIA IDC, ER^+^ 90% PR- HER2neu 2+ IHC, FISH negative primary (T1cN1M0), PD-L1 negative by 22C3. She underwent left-sided mastectomy and sentinel lymph node biopsy followed by adjuvant chemotherapy with dose-dense Adriamycin, cyclophosphamide, and paclitaxel. The patient received endocrine therapy with anastrozole from June 2007 to July 2014. Metastatic disease was diagnosed in August 2014 with biopsy-proven ER^+^ 95% PR 1% HER2neu- disease with bone, neck, and LN metastasis. She received first-line therapy with fulvestrant from August 2014 to April 2015 with best response as SD. She progressed in May 2015 with a new lytic bone lesion. She started on second-line therapy with letrozole and palbociclib from May 2015 to March 2017 with best response of SD. She progressed with multiple liver metastases (the largest measured 4.2 cm by 1.4 cm) on 4 March 2017. The patient enrolled in this study on 27 March 2017 and received exemestane plus pembrolizumab through late November 2017 with best response as PR and PFS of 33 weeks. Upon progression, patient continued to receive four additional lines of therapy, including capecitabine, exemestane and everolimus, eribulin, and as of July 2021 (data cut-off), nab-paclitaxel.

### 3.3. Safety

Grade 3 adverse events (AE) per CTCAE 4.0 were rash (5%), elevated liver enzymes (5%) and lymphopenia (5%) (Table 3). Grade 2 AEs were fatigue (35%), rash (15%), hot flashes (10%), insomnia (5%), headache (5%), pruritis (5%), arthralgia (5%), dry mouth (5%), localized edema (5%), cough (5%), hypertension (5%), and hypothyroidism (5%). Immune toxicity was consistent with known pembrolizumab toxicity, as listed in package insert.

### 3.4. Immune Biomarkers

A total of 14 (70%) patients had PD-L1 results by IHC using the 22C3 antibody, with six lacking available tissue for testing. Of those 14, three (21%) patients were PD-L1 positive (PD-L1 ≥ 1% by 22C3 IHC testing), and 11 (79%) patients were PD-L1 negative (PD-L1 < 1% by 22C3 IHC testing). Of the two patients with PR, both were negative for PD-L1. One patient with SD had 50% PD-L1 staining (H score = 70), one patient with PD had 2% PD-L1 staining (H score = 4), and one patient with PD had 1% PD-L1 staining (H score = 1). 

Stromal TILs (sTILs) were analyzed for nine patients with available H&E slides (Table 4). Of these patients, one PR patient had 5% sTILs (supraclavicular mass), two SD patients had 5–10% sTILs (breast and chest wall), one PD patient had 70% sTILs (axillary LN), and five PD patients had 2–10% sTILs (two skin, two liver, one chest wall).

## 4. Discussion

In the current study, the combination of AI and pembrolizumab was well tolerated with a modest CBR of 20% (95% CI 5.7, 43.7) and ORR of 10% (95% CI 0.3, 44.5). The immune-related AEs were consistent with known profiles of pembrolizumab. Our result is comparable with the single agent pembrolizumab historical data in HR^+^ BC. Rugo et al. studied a cohort of 25 patients with HR^+^ MBC and PD-L1 ≥ 1% by 22C3 IHC testing [20]. The incidence of treatment-related adverse events was 64%, with grade 1 or 2 nausea (20%) and fatigue (12%). Rugo et al. reported a single agent pembrolizumab response rate of 12% in a cohort of 25 patients with PD-L1^+^ HR^+^ MBC [21]. The combination of CTLA-4 inhibitor tremelimumab and exemestane showed 42% SD only in a cohort of 26 patients with heavily pretreated HR^+^ MBC [22]. The low immunogenicity of HR^+^ HER2^−^ MBC compared with TNBC may explain this modest clinical activity [23,24]. HR^+^ HER2^−^ breast cancer is well known for its lack of TIL and PD-L1 expression, hence considered “immune-cold tumors” [11]. Approximately 10−15% of HR^+^ HER2^−^ BC expresses PD-L1 detected by 22C3 antibody testing. Preclinical evidence has shown that high ER levels decrease PD-1/PD-L1 expression and CD8^+^ T cell infiltration by suppressing Th17 cell infiltration and IL-17 signal transduction in breast cancer [25]. Antiestrogen therapy with SERDs, particularly when administered in combination with anti-PD-L1 antibodies, acts to inhibit BC progression in part by blocking the expansion and mobilization of myeloid-derived stem cells (MDSC), suppress regulatory T cells (Tregs), and increase Th1 response [25]. These preclinical data indicate that SERDs in combination with ICI may be worth further testing in HR^+^ MBC. 

The modest clinical activity observed in the current and earlier immune checkpoint inhibitor trials indicates that novel combination therapy eliciting robust immune response to turn “immune-cold” tumors into “immune-hot” tumors is necessary to enhance the efficacy of checkpoint inhibitors in HR^+^ MBC. Modulation of the host immune and tumor microenvironment may be the key to effective immune responses in HR^+^ HER2^−^ MBC. In theory, a number of targeted therapies could act as an “immune-switch”, such as chemotherapy, radiation therapy, epigenetic targeting drugs, oncolytic viruses, anti-angiogenic therapies, immune cytokines, tumor vaccines, and cellular therapies [26]. 

CDK4/6 inhibitors have demonstrated PFS and OS benefit in HR^+^ HER2^−^ MBC. Beyond cell cycle suppression, immune-modulatory effects of CDK 4/6 inhibitors were well documented in both preclinical and clinical trial settings. Goel et al. demonstrated that CDK4/6 inhibition stimulates production of type III interferons, enhances tumor antigen presentation, and markedly suppresses the proliferation of regulator T cells [27]. CDK4/6i was also shown to increase tumor infiltration and activation of effector T cells and augment the response to PD-1 blockade in a novel ex vivo organotypic tumor spheroid culture system and in multiple in vivo murine syngeneic models by Deng et al. [28]. In a recent phase I trial, we tested the combination of AI, palbociclib, and pembrolizumab in patients with ER^+^ HER2^−^ MBC [29], with a CR of 31%. In our peripheral blood mononuclear cells (PBMCs) flow cytometry analysis, CDK4/6i showed immune-priming effect with significantly increased fractions of type 1 conventional dendritic cells (cDC1s) within circulating dendritic cells and decreased classical monocytes (cMO) within circulating monocytes only in patients treated with palbociclib [30]. To fully exploit the immune-priming effect of CDK4/6i, future confirmatory trials are needed.

The current study is limited by its small sample size and the trial design, which was a non-randomized, single-arm trial in endocrine-resistant patients who received prior lines of endocrine therapy and were unselected for PD-L1 expression. Future studies with larger patient cohorts and randomized designs are necessary to explore novel combinations with ICI in incurable HR^+^ HER2^−^ MBC patients. 

## 5. Conclusions

The combination of pembrolizumab and AI is well tolerated in PD-L1 unselected patients with HR^+^ HER2^−^ MBC. The modest clinical activity observed in this study indicates that novel combination therapies are required to boost immune-mediated responses and clinical benefit ratio and duration in HR^+^ MBC patients.

## Figures and Tables

**Table 1:** Patient characteristics and treatment variables (N = 20).

Characteristic	N (%)
Age (median, range)	62 (34–79)
Gender	
Female	20 (100%)
Race	
White	15 (75%)
Asian	3 (15%)
Pacific Islander	1 (5%)
Unknown	1 (5%)
Performance status	
0	8 (40%)
1	12 (60%)
Initial tumor stage	
Stage I	2 (10%)
Stage II	5 (25%)
Stage III	10 (50%)
Stage IV	3 (15%)
Initial histology grade	
Grade II	15 (75%)
Grade III	1 (5%)
Not done	1 (5%)
Unknown	3 (15%)
Prior surgery	17 (85%)
Prior radiation	12 (60%)
Lines of chemotherapy for MBC	
0	1 (5%)
1	3 (15%)
2	7 (35%)
3+	9 (45%)
Sites of metastasis	
Bone	18 (90%)
Liver	12 (60%)
Lung	4 (20%)
Brain	1 (5%)
Other *	11 (55%)
Prior CDK 4/6i	14 (70%)
Prior Fulvestrant	15 (75%)
Prior AI	14 (70%)
Prior Everolimus	7 (35%)

* Skin, chest wall, pleural effusion, pleural nodule, adrenal gland, bladder, colon, lymph nodes.

**Table 2:** Evaluable Response per RECIST 1.1 (N = 20).

Best Response	N (%)
PR	2 (10%)
SD	3 (15%)
PD	15 (75%)
CBR (6 months)	4 (20%) (95% CI 5.7, 43.7)
ORR (CR + PR)	2 (10%) (95% CI 0.3, 44.5)

PR, partial response; SD, stable disease; PD, progression of disease; CBR, clinical benefit rate; ORR, overall response rate.

**Table 3:** Adverse events with attributions in “Definite”, “Possible”, “Probable” (grade 2+ only, and for all courses) per CTCAE 4.0 (N = 20).

Adverse Event	Grade 2	Grade 3
All adverse events (worst grade per patient)	12	2
Rash	4 (20%)	1 (5%) *
Elevated AST/ALT	1 (5%)	1 (5%)
Lymphopenia		1 (5%) *
Fatigue	7 (35%)	
Hot flashes	2 (10%)	
Insomnia	1 (5%)	
Headache	1 (5%)	
Pruritus	1 (5%)	
Arthralgia	1 (5%)	
Dry mouth	1 (5%)	
Localized edema	1 (5%)	
Cough	1 (5%)	
Hypertension	1 (5%)	
Hypothyroidism	1 (5%)	

* Same patient.

**Table 4:** Tumor Immune Biomarker sTILs and PD-L1 with 22C3 antibody (N = 20).

Patient ID	Best Response	Tissue	% sTILs	PD-L1
COH-01	PD	NA	NA	NA
COH-02	PD	NA	NA	NA
COH-03	SD	NA	NA	NA
COH-04	PD	Skin	5%	Neg
COH-05	PD	Liver	10	1%
COH-06	SD	Breast	5%	Neg
COH-07	PD	Frontal tumor	NA	Neg
COH-08	PR	Breast	NA	Neg
COH-09	PD	Liver	NA	NA
COH-10	PR	Supraclavicular mass	5%	Neg
COH-11	PD	Liver	5%	Neg
COH-12	PD	Bone	NA	Neg
COH-13	SD	Chest wall	10%	50%
COH-14	PD	Axillary LN	70%	NA
COH-15	PD	Ovary	NA	2%
COH-16	PD	Skin	2%	Neg
COH-17	PD	LN	NA	NA
COH-18	PD	Liver	NA	Neg
COH-19	PD	Chest wall	10%	Neg
COH-20	PD	Liver	NA	Neg

sTILs, stromal tumor infiltrating lymphocytes; PR, partial response; SD, stable disease; PD, progression of disease; NA, not applicable; LN, lymph node; Pos, positive; Neg, negative.

## Data Availability

The data can be shared upon reasonable request.
